# A micro-bead device to explore *Plasmodium falciparum*-infected, spherocytic or aged red blood cells prone to mechanical retention by spleen endothelial slits

**DOI:** 10.1186/1475-2875-9-S2-O10

**Published:** 2010-10-20

**Authors:** Guillaume Deplaine, Innocent Safeukui, Fakhri Jeddi, François Lacoste, Valentine Brousse, Sylvie Perrot, Sylvestre Biligui, Micheline Guillotte, Corinne Guitton, Safi Dokmak, Béatrice Aussilhou, Alain Sauvanet, Anne Couvelard, François Paye, Marc Thellier, Dominique Mazier, Geneviève Milon, Narla Mohandas, Odile Mercereau Puijalon, Peter H David, Pierre A Buffet

**Affiliations:** 1 Institut Pasteur, Unité d'Immunologie Moléculaire des Parasites, Département de Parasitologie Mycologie, F- 75015 Paris, France; 2 CNRS, URA2581, Paris, France; 3 INSERM - UPMC (Paris 6 University) UMRs945, F-75013 Paris, France; 4 Fond Ackermann, Fondation de France; 5 Department of Pediatrics, Necker Hospital, AP-HP, F-75015 Paris, France; 6 Department of Parasitology, Pitié Salpétrière Hospital, AP-HP, F-75013 Paris, France; 7 Department of Haematology, Kremlin-Bicêtre Hospital, AP-HP, F-94270 Le Kremlin-Bicêtre, France; 8 Department of Surgery, Beaujon Hospital, AP-HP, F-92110 Clichy, France; 9 Department of Pathology, Beaujon Hospital, AP-HP, F-92110 Clichy, France; 10 Department of Surgery, Saint-Antoine Hospital, AP-HP, F-75018 Paris, France; 11 Institut Pasteur, mmunophysiologie et Parasitisme Intracellulaire, Département de Parasitologie Mycologie, F-75015 Paris, France; 12 New York Blood Centre, New York, NY 10065, USA

## 

Experimental tools to identify human red blood cells (RBC) prone to mechanical retention upstream from the spleen venous sinus inter-endothelial slits are currently suboptimal. We designed a micro-bead device mimicking the geometry of the human narrow and short inter-endothelial slits. Upon filtration through a mixture of 5-25 μm diameter micro-beads, *Plasmodium falciparum-hosting* RBC (Pf-RBC) were retained in a parasite developmental stage-dependent way, the retention rates of a subset of ring-RBC being similar in micro-beads and in isolated-perfused human spleens. We found that this retention might be linked principally to the reduced surface-area-to-volume ratio of *Pf-RBC.* Interestingly, other rigid RBC, such as heat-treated RBC, and RBC from hereditary spherocytosis patients were also retained in micro-beads without any hemolysis. Micro-beads allow (i) depletion of heterogeneous RBC population from its rigid-RBC subpopulation ii) characteriziation of distinct molecular signatures of rigid versus deformable RBC subpopulations. This simple method portends wide medical applications, such as improving the quality of stored RBC concentrates prior to transfusion.

